# Secondary Central Nervous System Demyelinating Disorders in the Elderly: A Narrative Review

**DOI:** 10.3390/healthcare11152126

**Published:** 2023-07-25

**Authors:** Christos Bakirtzis, Maria Lima, Sotiria Stavropoulou De Lorenzo, Artemios Artemiadis, Paschalis Theotokis, Evangelia Kesidou, Natalia Konstantinidou, Styliani-Aggeliki Sintila, Marina-Kleopatra Boziki, Dimitrios Parissis, Panagiotis Ioannidis, Theodoros Karapanayiotides, Georgios Hadjigeorgiou, Nikolaos Grigoriadis

**Affiliations:** 1 Second Department of Neurology, Aristotle University of Thessaloniki, GR-54124 Thessaloniki, Greece; marilima_1992@hotmail.com (M.L.); iradel7714@gmail.com (S.S.D.L.); ptheotokis@auth.gr (P.T.); kesidoue@auth.gr (E.K.); nataliak95@gmail.com (N.K.); linsintila@yahoo.gr (S.-A.S.); bozikim@auth.gr (M.-K.B.); dparisis@auth.gr (D.P.); ispanagi@auth.gr (P.I.); tkarapanayiotides@auth.gr (T.K.); ngrigoriadis@auth.gr (N.G.); 2 Faculty of Medicine, University of Cyprus, Nicosia CY-2029, Cyprus; artemiadis.artemios@ucy.ac.cy (A.A.); hadjigeorgiou.georgios@ucy.ac.cy (G.H.)

**Keywords:** demyelination, postinfectious, metabolic, nutritional, radiation-induced, drug-induced, paraneoplastic

## Abstract

Secondary demyelinating diseases comprise a wide spectrum group of pathological conditions and may either be attributed to a disorder primarily affecting the neurons or axons, followed by demyelination, or to an underlying condition leading to secondary damage of the myelin sheath. In the elderly, primary demyelinating diseases of the central nervous system (CNS), such as multiple sclerosis, are relatively uncommon. However, secondary causes of CNS demyelination may often occur and in this case, extensive diagnostic workup is usually needed. Infectious, postinfectious, or postvaccinal demyelination may be observed, attributed to age-related alterations of the immune system in this population. Osmotic disturbances and nutritional deficiencies, more commonly observed in the elderly, may lead to conditions such as pontine/extrapontine myelinolysis, Wernicke encephalopathy, and demyelination of the posterior columns of the spinal cord. The prevalence of malignancies is higher in the elderly, sometimes leading to radiation-induced, immunotherapy-related, or paraneoplastic CNS demyelination. This review intends to aid clinical neurologists in broadening their diagnostic approach to secondary CNS demyelinating diseases in the elderly. Common clinical conditions leading to secondary demyelination and their clinical manifestations are summarized here, while the current knowledge of the underlying pathophysiological mechanisms is additionally presented.

## 1. Introduction

The myelin sheath is an important protective layer coating the axon of the neuron. The fundamental role of myelin is to preserve normal neuronal function. Myelin destruction, known as demyelination, is a common pathological finding which leads to a wide spectrum of neurological manifestations. Demyelination may be acute or chronic, occurring either in the central or the peripheral nervous system. Regarding central nervous system (CNS) demyelination, common primary demyelinating diseases, such as multiple sclerosis (MS) are characterized by the predominant damage of the myelin sheath, often accompanied by axonal injury [1]. MS is one of the most common causes of non-traumatic neurological disability in young people [2]. Additionally, other inflammatory demyelinating disorders, such as anti-MOG associated disease and neuromyelitis optica spectrum disorders (NMOSD), are mainly diagnosed in early and middle adulthood [3]. The incidence of the aforementioned neurological entities is very low in the elderly population, above 60 years old. Therefore, when the first demyelinating event occurs in individuals older than 60 years old, other causes which secondarily affect myelin should be extensively investigated.

Secondary demyelinating diseases may either be attributed to a disorder primarily affecting the neurons or axons, causing demyelination, or to an underlying condition leading to secondary damage of the myelin sheath [4]. Numerous conditions and diseases with characteristic radiological patterns may give rise to secondary demyelinating diseases. In the current review, we aim to summarize and present common causes of CNS secondary demyelinating diseases, including infectious, postinfectious, postvaccinal, metabolic, nutritional, toxic, iatrogenic, and paraneoplastic syndromes.

## 2. Infectious, Postinfectious, and Postvaccinal Causes of CNS Demyelination

### 2.1. Age-Related Alternations of the Immune System in Elderly Individuals

The term immunosenescence refers to the age-related qualitative and quantitative decline of the immune system, affecting both innate and adaptive immunity [5]. Immunosenescence contributes to an increased incidence of severe infections, a decreased response to immunization with vaccination and possibly to an increased incidence of malignancies in the elderly [6,7].

Considering innate immunity, the decreased efficacy of epithelial barriers, including the skin, the lungs, and the gastrointestinal tract (GIT), facilitates pathogen invasion [8]. Additionally, changes in the number and functions of crucial cellular components, such as neutrophils, macrophages, and natural killer (NK) cells facilitate the weakening of the immune system [9]. Neutrophils offer protection against bacterial and fungal pathogens during acute inflammation. Although their number, chemotactic function, and adhesion capacity remain unaffected with time, a significant decrease in their ability to phagocytose opsonized bacteria has been observed [10]. Changes in the functions of macrophages have also been observed in older individuals; their decreased ability to produce chemokines disrupts the normal communication between innate and adaptive immunity and is associated with persistent infections [11]. Cytotoxic NK cells are responsible for the recognition of host cells infected by viral agents. In contrast to the changes occurring in other cells of the innate immune system, a significant increase in the number of NK cells has been identified during ageing [12]. However, it is characterized by a decrease in cytotoxicity and in the ability to produce interferon-γ [13].

Regarding adaptive immunity, T- and B-lymphocytes demonstrate significant changes during ageing. The involution of the thymus starts early in life and is completed by the age of 40–50 years. Involution leads to a decrease in the number and diversity of naïve CD4+, CD8+, and T regulatory cells exiting the thymus and is particularly seen in individuals above the age of 70 years old [14,15]. These naïve cells exhibit decreased proliferation capacity and increased susceptibility to activation-induced cell death [16]. Moreover, the accumulation of CD8+ effector and memory cells, as well as the decrease of nonregulatory CD8 + CD4 + RO + CD25 + T cells ultimately lead to reduced humoral responses to vaccination [17,18]. Another important factor associated with T-cell aging is the presence of latent viral infections, caused by frequently encountered pathogens including cytomegalovirus (CMV), Epstein–Barr virus (EBV), human immunodeficiency virus (HIV), herpes simplex virus (HSV), and John Cunningham virus (JCV). This lifelong continuous stimulation of the immune system, leads to the development of a persistent subclinical inflammatory status, known as inflammaging [5]. Chronic inflammation has also been associated with the induction of neurodegeneration [19].

Similar changes to those occurring in T-lymphocytes are also seen in B-lymphocytes; the number of naïve B-cells decreases, whereas memory cells progressively accumulate due to their decreased susceptibility to apoptosis, leaving the total number of B cells in the peripheral blood unaffected [20]. Regarding B memory cells, there is a shift in antibody isotype production from IgG to IgM with time, therefore reducing long-term response to vaccination [21]. Furthermore, a decrease in B-cell expansion and differentiation, as well as in their stimulation by dendritic cells leads to decreased B-cell responses [22].

### 2.2. Demyelination Due to Infection

#### 2.2.1. Progressive Multifocal Encephalopathy

Progressive multifocal encephalopathy (PML) is a rare, but life-threatening disease of the CNS, caused by the JCV, a double-stranded DNA virus which belongs to the Polyomaviridae family and was first isolated in 1971 [23]. According to studies, JCV DNA can be detected in up to 60% of the population by the age of 70 [24]. The virus establishes a latent infection in immunocompetent individuals, who remain asymptomatic, whereas immunosuppressed patients, especially those with prolonged CD4+ T-cell depletion, may develop PML due to viral reactivation and uncontrolled replication. The most common underlying condition associated with PML is HIV infection, followed by lymphoproliferative disease and drug-induced immunosuppression [25]. Nevertheless, cases of PML in elderly people without any apparent immunosuppression have been reported; it is suggested that immunosenescence may be the underlying pathophysiological mechanism for their vulnerability to JCV [26].

Nowadays, PML is subdivided into three distinct clinical types: classical, inflammatory, and iatrogenic. Common clinical manifestations of classical PML are cognitive deficits, sensorimotor deficits, ataxia, aphasia, cortical visual disturbances, and sometimes convulsions. Histopathological findings reveal enlarged oligodendrocytes and “bizarre” astrocytes in the presence of multifocal demyelination [27]. Neuroimaging with brain magnetic resonance imaging (MRI) shows a characteristic pattern with T2 hyperintensities and subcortical U-fiber involvement, as well as diffusion restriction [28]. Paradoxically, inflammatory PML or “PML-IRIS” (immune reconstitution inflammatory syndrome) develops after immune reconstitution in previously immunocompromised patients, for example after treatment administration in HIV infection or treatment discontinuation in drug-induced immunosuppression [29]. PML-IRIS has been associated with specific MRI patterns, different from those seen in other types of PML, suggesting perivascular inflammation [30,31,32,33]. The most recently defined type of PML is the iatrogenic form which is caused by drug-induced immunosuppression and was first described in patients receiving rituximab, but nowadays, it is most frequently seen in MS patients receiving natalizumab [34,35]. Additionally, PML-IRIS may develop within the first few weeks after natalizumab discontinuation and plasma exchange (PLEX) which is frequently used for faster drug clearance [36]. Therefore, iatrogenic PML may present either as classic or as inflammatory and MRI reveals a “milky way appearance”. For this reason, natalizumab discontinuation should be accompanied by close patient monitoring including neuroimaging [37,38].

Currently, there is no available treatment for PML. Immune reconstitution with HAART administration in HIV+ patients, drug discontinuation, or PLEX in natalizumab-associated PML is suggested. Studies examining the use of Maraviroc, a CCRS antagonist which limits CCRS+ T-cells trafficking into CNS, revealed mixed results [39]. Interestingly, the administration of immune checkpoint inhibitors (ICIs) showed promising results. However, ICIs are associated with serious side effects and high morbidity rates [39].

#### 2.2.2. Granule Cell Neuropathy

Despite PML, reactivation of a mutated JCV may affect the granule cell neurons in the cerebellum of immunocompromised individuals, leading to the development of granule cell neuropathy, a condition even rarer than PML. Patients present with progressive, subacute gait disturbances, dysarthria, ataxia, and nystagmus. MRI findings include bilateral, diffuse, symmetric cerebellar atrophy with or without white matter changes in the pons and middle cerebellar peduncles. Similar to PML, there is no available treatment for granule cell neuropathy at the moment, and patients receive only supportive management for their neurological symptoms [40].

#### 2.2.3. Tabes Dorsalis

Syphilis, a sexually transmitted disease caused by the bacterium Treponema pallidum, affects the spinal cord by causing demyelination of the posterior column and posterior roots of the spinal cord [41,42]. The pathogenesis of the disease is based on large myelinated fiber invasion by Treponema pallidum, leading to inflammation and eventual nerve degeneration. The inflammation is further induced by a perivascular inflammatory response against the treponeme along with gummas, where macrophages and T-helper cells produce proinflammatory cytokines [43].

Although bacterial CNS invasion seems to occur early in the course of the disease, patients exhibit clinical manifestations 15- 20 years after primary infection, at the tertiary stage of the disease. Therefore, tabes dorsalis appears later in life and can also affect elderly people [44]. Patients may present with spastic paraparesis and autonomic disturbances. Clinical signs in tabes dorsalis include areflexia, multimodality sensory loss, optic atrophy, and Argyll Robertson pupil [45]. Laboratory investigation with treponemal serology testing (VDRL) for the detection of antibodies against Treponema pallidum in the cerebrospinal fluid (CSF) should be performed [46]. MRI findings with gadolinium enhancement include the “flip-flop sign” which is caused by the presence of edema and hyperintensities in the affected area of the spinal cord, and a “candle guttering appearance” due to a peripheral band-like appearance [47,48].

Early treatment administration is crucial for symptom regression and disease progression arrest since delayed treatment initiation can only halt disease progression. Penicillin G administered intravenously is the first line of treatment with or without the use of adjuncts, such as probenecid, for all patients. Unfortunately, relapses are commonly seen in HIV+ patients and therefore, treatment should be repeated [45]. In HIV- patients with penicillin allergy, doxycycline can be used instead. Strong analgesics including opiates, but also antiepileptic medications such as valproate, can be used for the management of pain crises [49,50].

### 2.3. Postvaccinal Demyelination

Both postvaccinal and postinfectious demyelination are caused by the same underlying pathophysiological mechanism. The term “molecular mimicry” refers to the similarities between the host and the microbial proteins of the pathogen entering the body, resulting in a cross-reacting immune response and the formation of autoantibodies, which sometimes target the myelin sheath, causing demyelination. Both vaccines and infections can act as immune activators and may lead to demyelination [51]. Usually, clinical features develop 2–3 weeks after vaccination or symptomatic infection. According to studies, both the inactivated and the live-attenuated vaccines against influenza virus, the recombinant DNA technology vaccine against hepatitis B virus, the live-attenuated rubella virus vaccine, and the live-attenuated rabies vaccine have been associated with postvaccinal demyelination [52]. Recently, several cases of postvaccinal multifocal demyelination following the vaccination with the various vaccine types against SARS-CoV-2 have also been described, even in elderly people [53]. However, the incidence of postvaccinal demyelination in the elderly population has not been estimated yet. Antibodies against the myeline oligodendrocyte glycoprotein (MOG) and aquaporin 4 (AQP4) which are associated with CNS demyelination have been detected in some patients [54].

### 2.4. Postinfectious Demyelination

#### 2.4.1. Acute Disseminated Encephalomyelitis

Acute disseminated encephalomyelitis (ADEM) is a monophasic, postinfectious or postvaccinal demyelinating disease in most cases, mainly seen in children; however, several cases have been reported in elderly people [55,56]. After infection remission or vaccine immunization, individuals with ADEM usually present with moderate-to-severe meningism, headache, fever, and altered mental status, but also focal neurological deficits. Histopathological findings reveal inflammation limited to perivascular mononuclear cells with or without microglia located at the edge of the lesion. Multifocal periventricular lesions are found in the subcortical white matter, as well as the deep grey matter, and sometimes the spinal cord [57]. Radiological imaging with MRI shows periventricular or diffuse edema with or without multifocal lesions, in the absence of necrosis or atrophy. MOG-IgG is detected in approximately half of the patients [58], whereas intrathecal IgG production with oligoclonal bands (OCBs) might be seen [59].

For the treatment of ADEM, intravenous steroids are often used. In severe or refractory cases, PLEX with or without corticosteroid administration may be required. Μost of the patients achieve complete remission. In case of relapsing disease, the differential diagnosis should include conditions such as MS and NMO that may rarely present with an ADEM-like onset [60].

#### 2.4.2. Postinfectious Encephalitis

Postinfectious encephalitis is usually attributed to a recent herpes Simplex-1 virus (HSV) infection [61]. The N-methyl-D-aspartate receptor (NMDAR) antibody is highly associated with postinfectious HSV encephalitis and has been detected in a significant number of cases, indicating an underlying auto-immune mechanism [62]. NMDA receptors are located in the myelinating processes of oligodendrocytes; anti-NMDAR leads to demyelination [63]. Other viruses capable of triggering NMDAR encephalitis are VZV and EBV [64]. Lately, cases of postinfectious encephalitis following COVID-19 infection, by the recently identified SARS-CoV-2 virus have also been reported [65].

Clinical manifestations in aged people are usually predominated by neuropsychiatric symptoms. Bilateral MRI hyperintensities involving the cortical and subcortical regions of the frontal and temporal lobes, as well as the insula, are frequently seen. Additional gyral swelling with or without enhancement might be visualized, whereas after 48 h, petechial hemorrhages might be present [66]. Immunotherapy with high doses of corticosteroids, IVIG, or PLEX followed by immunosuppressive treatment with rituximab, mycophenolate mofetil, or cyclophosphamide, is frequently used [61].

## 3. Metabolic and Nutritional Causes of CNS Demyelination

### 3.1. Central Pontine and Extrapontine Myelinolysis

Central pontine myelinolysis (CPM) is a monophasic demyelinating disease affecting the pons with or without extrapontine involvement [67]. The most common cause of CPM is the rapid correction of hyponatremia, usually occurring in malnourished individuals due to chronic alcohol misuse or severe illness and it was first described by Adams, in 1959 [68]. In addition to hyponatremia, any hyper- or hypo-osmolar state followed by rapid correction can lead to osmotic demyelinating syndrome. Aggressive correction of hyperglycemia and hypokalemia can also lead to osmotic demyelination in pons or extrapontine regions, such as the cerebellum [67].

Hyponatremia (serum sodium < 136 mEq/L) is an electrolyte disturbance that is frequently encountered in the elderly, particularly during summer due to increased water consumption followed by increased sodium loss [69]. Several factors associated with older age contribute to the development of hyponatremia including the increased secretion of antidiuretic hormone (SIADH) in elderly individuals [70]. Other endocrinopathies such as hypothyroidism whose prevalence increases with age, and rarely hypopituitarism and Addison’s disease can also cause hyponatremia [71]. In addition, the use of certain medications such as thiazide diuretics, serotonin-reuptake inhibitors, serotonin and norepinephrine reuptake inhibitors, and non-steroidal anti-inflammatory drugs may provoke hyponatremia in the elderly [72,73]. The coexistence of comorbidities including diabetes mellitus (DM), heart failure, liver diseases, and malignancy often contribute to the development of sodium abnormalities [74]. Additionally, hypertension is associated with low sodium levels since patients are often advised to follow a strict low-sodium diet. Finally, the age-related physiologic decrease in the glomerular filtration rate (GFR) induces water retention [75].

Hyponatremia decreases serum tonicity which leads extracellular water to shift into cells, leading to cerebral edema. The brain uses several adaptive mechanisms to withstand these changes, including the displacement of water into the CSF, the removal of extracellular solutes and water via ion channels, and the efflux of organic osmolytes and water. Therefore, in case of rapid correction, the brain is unable to recapture these lost osmolytes and becomes dehydrated with subsequent white matter demyelination. Histopathological findings reveal demyelination with cavitation. Although there is oligodendrocyte and myelin degeneration, neurons and axons remain intact [67].

Clinical features include bulbar weakness and rapidly progressing to quadriparesis within days. Altered consciousness levels including confusion, lethargy, and coma are also common. Neuroimaging with MRI shows demyelination in central pons with extrapontine involvement in in about 10% of patients including the subcortical white matter, cerebellum, lateral geniculate body, basal ganglia, thalamus, and internal capsule [76]. Preventative measures should be taken in case of hyponatremia and the rate of correction should not exceed 8–12 mEq/L/day (<12 mmol/L/day) [77].

### 3.2. Nutritional Deficiencies

A wide range of factors including chronic illness and iatrogenic interventions may lead to nutritional deficiencies and/or malnutrition in the elderly. Conditions such as atrophic gastritis, decreased GIT mobility, inflammatory bowel disease, decreased gastric acid secretion, and GIT surgery contribute to decreased nutrient absorption [78]. Impaired absorption can also be caused by the use of certain medications, such as protein pump inhibitors [79]. Older individuals have decreased skeletal muscle mass, and therefore, decreased water storage capacity. Fluid and electrolyte disturbances may also be attributed to the decreased GFR due to ageing [80]. The prevalence of malnutrition is estimated at 5–10% in independently living elderly individuals, 35–65% in hospitalized patients and is further increased in patients living in nursing homes [81].

#### 3.2.1. Vitamin B12 Deficiency

Vitamin B12 dissociates in the stomach under the influence of pepsin and gastric acid, binds to the intrinsic factor, and gets absorbed in the terminal ileum. Therefore, malabsorption is an important causative factor of this deficiency [82]. Conditions including atrophic gastritis, decreased GIT mobility, inflammatory bowel disease (IBD), decreased gastric acid secretion, and GIT surgery contribute to decreased nutrient absorption. Impaired absorption can also be caused by the use of certain medications, for example, protein pump inhibitors (PPIs), histamine_2_-receptor antagonists, and metformin [83,84,85,86,87]. A prolonged decrease in vitamin B12 levels leads to decreased myelin protein methylation, decreased vacuolization of white matter, inappropriate growth of oligodendrocytes, and abnormal myelin production [88].The prevalence of vitamin B12 deficiency is about 10–15% in individuals over 60 years old [89].

Neurological complications usually appear insidiously. Patients may develop subacute combined degeneration with or without peripheral neuropathy and vice versa, optic neuropathy, neuropsychiatric symptoms, and orthostatic hypotension [90]. Subacute combined degeneration is a form of myelopathy with pyramidal and posterior column signs, caused by spinal cord demyelination with axonal loss which might be accompanied by peripheral neuropathy due to large nerve fiber demyelination. Additionally, cortical white matter involvement might occur. Subacute combined degeneration mostly occurs in patients 50–80 years old [89]. Neuroimaging with MRI scans may reveal spinal cord and white matter hyperintensities in T2-weightned images. Laboratory testing might reveal megaloblastic anemia, pancytopenia, and macrocytosis. One third of patients with vitamin B12 have increased serum homocysteine levels which can be used as a marker after treatment initiation [87,89]. A series of laboratory and imaging tests need to be performed in order to identify the potential presence of an underlying disease leading to vitamin B12 deficiency.

Treatment includes both vitamin supplementation and management of neurological complications, as well as that of the underlying disease, if present. Early treatment initiation is crucial since delayed administration is highly associated with residual deficits. In patients with malabsorption, intramuscular injections are required and the need for life-long treatment is frequently seen [91].

#### 3.2.2. Folic Acid Deficiency

Folic acid (folate) deficiency may develop alone or together with vitamin B12 deficiency. Folic acid is an important compound for the synthesis of nucleic acids and the conversion of homocysteine to methionine [92]. Drug-induced folic acid deficiency is associated with the use of trimethoprim, methotrexate, and triamterene [93]. The prevalence of folic acid deficiency is 14% in 50–60 year-old individuals and 23% in those >80 years old [94].

Serum folic acid levels drop within the first three weeks after intake decreases. However, neurological manifestations take months to develop. Typical clinical features arise from malfunction in the spinal cord, peripheral nerves or both. Laboratory results reveal decreased serum folic acid and increased homocysteine levels. MRI findings include T2 hyperintensities in the spinal cord [93]. Folic acid supplementation can be given orally, but parenteral administration is required for patients with malabsorption [95].

#### 3.2.3. Thiamine Deficiency

Vitamin B1, known as thiamine, is a water-soluble vitamin that functions as a coenzyme in the metabolism of carbohydrates, lipids, and amino acids [96]. During states of increased metabolic intake, underlying malignancy, or chronic illness the demand is increased. The aforementioned conditions, as well as increased thiamine loss due to hyperemesis or kidney failure, may lead to thiamine deficiency in the elderly [97].

Vitamin B1 deficiency can affect both the CNS and PNS. Clinical manifestations include Wernicke encephalopathy, usually progressing to Korsakoff syndrome, dry or wet Beriberi, and rapidly progressive axonal sensorimotor peripheral neuropathy [97,98,99]. Wernicke encephalopathy typically presents with ophthalmoparesis accompanied by diplopia and nystagmus, ataxia, and altered conscious level. Approximately 80% of the patients who recover from Wernicke encephalopathy develop Korsakoff syndrome, presenting with severe anterograde and retrograde amnesia [99]. MRI in patients with Wernicke encephalopathy reveals symmetric T2-hyperintensities located periventricularly, but also involving the mammillary bodies, thalami, tectal plate, and periaqueductal grey matter [100,101].

For the treatment of Wernicke encephalopathy, high doses of thiamine are required. Preventive thiamine administration is indicated in high-risk patients before the administration of glucose or total parenteral nutrition [102].

#### 3.2.4. Copper Deficiency

Copper deficiency in elderly people is often caused by excessive zinc ingestion and can lead to several neurological complications [103]. Patients may present with demyelinating conditions of the CNS and PNS such as myelopathy, neuropathy, myeloneuropathy, and optic neuropathy [104]. Laboratory results in cases of copper deficiency typically show anemia and neutropenia, accompanied by decreased serum copper and ceruloplasmin levels, as well as decreased copper excretion in 24-h urine collection [105]. In case of copper deficiency due to excessive zinc ingestion, increased serum zinc and zinc excretion in urine might be found [103]. Treatment of these conditions requires oral copper supplementation which improves hematological abnormalities. However, despite vitamin supplementation, patients usually present residual neurological deficits [105].

#### 3.2.5. Other Nutritional Deficiencies

Other vitamin deficiencies causing neurological complications due to CNS demyelination are vitamin E, and niacin deficiencies. Vitamin E deficiency in elderly people may be caused by chronic cholestasis, malabsorption, and pancreatic insufficiency [106]. Clinical manifestations include symptoms attributed to damage in the spinal cord, ataxia, ophthalmoplegia, and dystonia. [107,108]. Niacin deficiency may cause encephalopathy and neuropathy. The presence of niacin metabolites found in urine can be used to confirm the diagnosis [109].

### 3.3. Marchiafava–Bignami Disease

Corpus callosum demyelination, also known as Marchiafava–Bignami disease, is another rare demyelinating disorder usually seen in alcoholic patients. The exact pathophysiological mechanism has not been fully understood yet; however, excessive alcohol consumption and vitamin B complex deficiency seem to play a vital role in the development of the disease. The onset of the disease may be acute, presenting with personality changes resembling frontal lobe syndrome and rapidly progressing to death, or subacute characterized by left-sided apraxia and hemialexia without agraphia [110]. Histopathological findings show demyelination in central regions of the corpus callosum, followed by necrosis, hemorrhage, and cavitation. In the early course of the disease, callosal enhancement may be observed in brain MRI scans, while in the later stages, callosal atrophy is typically found. Treatment includes alcohol cessation with additional thiamine and vitamin B complex supplementation [111].

## 4. Iatrogenic Causes of Secondary CNS Demyelinating Diseases

### 4.1. Radiation

The incidence of malignancy increases with age. Frequently, elderly cancer patients are ineligible for surgery due to the coexistence of various comorbidities, and therefore, less invasive treatment strategies are recommended. Radiation with or without the use of adjuvant chemotherapy is a typical alternative treatment option. However, radiation tolerance is decreased in older individuals in comparison with younger patients and may lead to radiation toxicity even with low doses of radiation. [112].

The administration of radiation may cause demyelination of the CNS, most often in the spinal cord and particularly in the cervical cord which is considered more radiosensitive [113]. Radiation-induced damage in the spinal cord usually develops within six months to two years after initial treatment and may be caused by misalignment and calculation errors. Risk factors for the development of postradiation spinal cord injury include higher total dose, dose per fraction >200cGy, whole-organ radiation, increased target volume, and the presence of comorbidities such as hypertension and diabetes mellitus. Particularly in the spinal cord, the target dose should not exceed 54 Gy [114].

Postradiation demyelination of the brain, known as radiation-induced leukoencephalopathy, might also occur within 6 to 8 months after initial therapy. MRI findings are characterized by T2-hyperintensities without enhancement or mass effect. After whole-brain radiation therapy, there is diffuse and symmetric involvement, with relative white matter and corpus callosum sparing. Initially, white matter changes are seen periventricularly. However, white matter changes become diffuse and cerebral atrophy also becomes evident within months or years [115]. Modifications in the protocols used for the treatment of brain tumors have significantly decreased the incidence of this potentially life-threatening complication [113,116]. Nevertheless, elderly patients receiving extended field irradiation therapy frequently develop postradiation encephalopathy, characterized by cognitive deficits and brain atrophy [116].

### 4.2. Anti-TNF Spectrum Agents

Tumor necrosis factor-alpha (TNF-α) is a proinflammatory cytokine capable of altering immune cell proliferation and survival, thus playing a critical role in inflammation and auto-immunity. In specific murine models, TNF-α decreased T-cell proliferation [117]. Increased TNF-α levels in patients with autoimmune disease led to the development of TNF-α inhibitors, such as infliximab and etanercept, which are very efficient in the management of rheumatoid arthritis, Crohn’s disease, ulcerative colitis, and other autoimmune disorders [118].

Although anti-TNFα side effects are relatively rare, their administration might cause neurotoxicity, particularly associated with the development of demyelinating disease [119]. It has been suggested that anti-TNFα administration increases the survival of specific T-cells targeting myelin, leading to demyelination [120]. Discontinuation rates of anti-TNFa inhibitors are increased in elderly populations, due to neurotoxic events [121,122].

Neurological side effects caused by demyelination encompass a wide spectrum of diseases with CNS and PNS involvement, ranging from the development of MS or MS- like demyelinating syndromes, Guillain–Barre syndrome, Miller–Fisher syndrome, and peripheral neuropathies [123,124,125]. MRI usually reveals T2-hyperintensities without gadolinium enhancement in T1 sequences. The administration of anti-TNFα in patients with a predisposition to demyelinating disease increases the risk up to 30% [126]. Treatment of these side effects is based on anti-TNF discontinuation and management of the underlying disease [127].

### 4.3. Immune Checkpoint Inhibitors

Immunotherapy with immune checkpoint inhibitors (ICIs) seems to be effective in the treatment of highly proliferative malignancies. ICIs promote the activation and proliferation of T-cells, but also host immunosurveillance, inducing malignant cell destruction by the host [128].

ICIs are currently used in the treatment of more than twenty different types of malignancy. The increased incidence of malignancy in elderly patients who are ineligible for chemotherapy makes ICIs a useful alternative treatment option since they are better tolerated in comparison with chemotherapy. However, elderly patients might present with a different profile of adverse events than young patients [129].

Neurological adverse events constitute of acute demyelinating encephalitis, subacute tumefactive demyelination, and various symptoms from the PNS such as neuropathies and myopathies [130]. Brain MRI usually reveals enhancing lesions in the cerebrum, cerebellum, brainstem, and sometimes the optic sheath as well [131]. Corticosteroid administration is used as a first line treatment followed by plasmapheresis or IVIG in case of refractory cases [132].

### 4.4. Cyclosporine-A

Cyclosporine-A (CsA) is a calcineurin inhibitor used preventively in prophylaxis against graft rejection after organ transplantation, but also in the treatment of diseases such as rheumatoid arthritis. Since CsA does not suppress bone marrow, it is widely used after liver, kidney, heart, and lung transplantations [133]. CsA is a substrate of both CYP3A and P-glycoprotein which are important for the elimination of many frequently administrated drugs. Since polypharmacy is commonly seen in elderly patients due to the increased burden of comorbidities in older age, drug interactions and related toxicities should be carefully evaluated. Regarding the occurrence of adverse events in old patients receiving CsA, the rates are increased compared with younger patients, particularly affecting renal function [134].

Cyclosporine-induced neurotoxicity affects both the CNS and PNS, giving rise to a wide range of neurological symptoms that are usually reversible after treatment discontinuation or dosage decrease. Neurotoxicity is associated with high dosage even if it is within the therapeutic range, intravenous administration, concomitant P450 inhibitors use, liver and kidney transplantation due to decreased metabolism and excretion of the drug leading to its accumulation and toxicity [135,136]. Clinical features include headaches, parkinsonism, occipital seizures, intracranial hemorrhage, aphasia, altered mental status parkinsonism, pseudobulbar palsy, posterior reversible encephalopathy syndrome and motor deficits, and peripheral neuropathy [137].

Radiological findings in MRI scans demonstrate T2 hyperintensities located in the cerebral cortex, juxtacortical white matter, and diffuse or focal swelling [138]. Histopathological findings include demyelination, astrocytosis, neuronal and glial swelling, focal necrosis, infiltration of monocytes and eosinophils, and edema [139].

## 5. Paraneoplastic Causes of CNS Demyelination

Paraneoplastic neurological syndromes present as remote effects of malignancy, in the absence of either direct tumor invasion by metastatic disease or indirect involvement caused by coagulopathy and treatment-related toxicity [140]. According to a nine-year population-based study, the median age of diagnosis of paraneoplastic neurological syndromes is 68 years old [141]. The most highly associated neoplasms with the development of paraneoplastic neurological syndromes are small-cell lung carcinomas (SCLC), including breast and ovarian carcinomas, seminomas, and lymphoproliferative diseases, usually Hodgkin’s lymphoma [140].

The pathogenesis of these syndromes relies on a vigorous immune response against antigens expressed in the tumor leading to the formation of anti-neuronal autoantibodies targeting either intracellular neuronal proteins or extracellular, either neuronal cell surface or synaptic proteins found in the CNS, PNS, autonomic nervous system, neuromuscular junctions, and muscles. Antibodies targeting extracellular neuronal proteins cause internalization of neuronal receptors, directly affecting neuronal function. The exact pathogenetic mechanism of antibodies targeting intracellular antigens remains unknown. Therefore, currently, the antibodies targeting intracellular proteins are not considered to be pathogenic [142].

Detection of a specific high-risk anti-neuronal antibody in a patient presenting with a high-risk phenotype for paraneoplastic syndrome indicates that the presence of an underlying malignancy is highly probable and thorough investigation with the use of whole-body positron emission tomography (PET) and CT is required. Smoking and older age are considered as important risk factors for paraneoplastic disease [140].

Treatment of paraneoplastic neurological syndromes requires concomitant management of the tumor and immunotherapy administration for the treatment of the neurological symptoms. First-line immunotherapy used for the induction of remission includes corticosteroids, IVIG, PLEX, and cyclophosphamide administration. For the maintenance of remission, long-term immunosuppression is required using azathioprine or rituximab administration [143].

### Paraneoplastic Encephalitis

Limbic encephalitis selectively affects the limbic system. Patients initially exhibit personality changes followed by memory loss and seizures [144]. Neuroimaging with MRI often demonstrates temporal lobe abnormalities. Several anti-neuronal antibodies have been detected in patients with limbic encephalitis including anti-Hu, anti-VGKA, and anti-NMDAR. The most highly associated underlying malignancy is SCLC [141,142].

Brainstem encephalitis is characterized by insidious onset. Patients present with nystagmus, ataxia, parkinsonism, and several cranial nerve neuropathies. Anti-Ma1 and anti-Ma2 antibodies have been detected in a large number of patients. The coexistence of both antibodies is seen in the presence of several underlying malignancies, and it is associated with worse neurological prognosis [145].

In MRI scans, paraneoplastic demyelination may additionally present as a solitary cerebral tumefactive lesion with gadolinium enhancement and minimal edema. Brain biopsy is often performed in order to enable the differential diagnosis from gliomas [146,147].

## 6. Discussion

A plethora of conditions may be the underlying cause of secondary CNS demyelinating disorders. In most cases, the damage is not restricted to the myelin sheath as neurons and/or axons are also impaired. This neurodegenerative component may sometimes minimally contribute to the initial clinical presentation; however, it may significantly affect the long-term outcome of these disorders. For example, treatment with radiation in primary brain tumors may lead to progressive cognitive dysfunction due to axonal loss [148], while oxidative stress and astrocyte dysfunction caused by thiamin deficiency may catalyze various neurodegenerative processes, accelerating the rates of brain atrophy [149]. Nevertheless, CNS demyelination seems to play a key role in the clinical expression of all the aforementioned disorders (Table 1). Interestingly, myelin loss has also been observed in cerebrovascular and neurodegenerative diseases, such as amyloid angiopathy and Alzheimer’s, however its contribution to the clinical manifestations is still under investigation [150,151].

**Figure 1:**
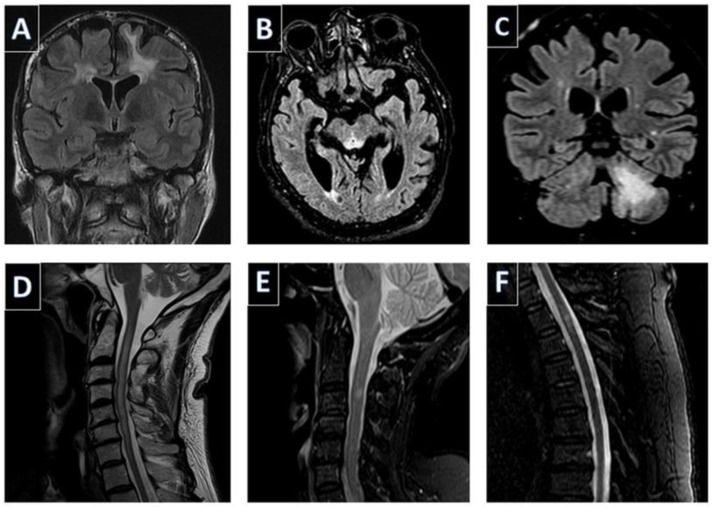
Representative images from brain (**A**–**C**) and spinal cord (**D**–**F**) MRI scans in aged people with secondary demyelination. (**A**) Coronal FLAIR image demonstrating diffuse T2 hyperintensities in a 68-year-old female with postradiation demyelination. (**B**) Axial FLAIR image demonstrating T2 hyperintensity in the periaqueductal area, in a 74-year-old male with Wernicke encephalopathy. (**C**) Coronal FLAIR image demonstrating a diffuse T2 lesion in the cerebellum, in a 71-year-old female with progressive multifocal leukoencephalopathy. (**D**) Sagittal T2-weighted image of the cervical spine demonstrates a postinfectious longitudinal extensive myelitis in a 65-year-old male. (**E**) Sagittal T2-weighted short-tau inversion recovery image of the cervical spine of a 66-year-old female with myelitis attributed to anti-TNF agents. (**F**) Sagittal T2-weighted image revealing a T2 lesion in the upper thoracic spinal cord of a 67-year-old female with myelitis attributed to cyclosporin.

MRI is widely used in clinical practice and therefore, it is the most common tool used in the detection of CNS demyelination. The application of advanced neuroimaging techniques is currently being further investigated in order to facilitate the differentiation between several conditions characterized by T2 hyperintensities. The most common example of the research with regards to advanced neuroimaging and CNS demyelination is the case of MS. Misdiagnosis of MS is frequently encountered in clinical practice and is often attributed to the misinterpretation of radiologic findings [152]. To address this matter, the radiologic criteria for the diagnosis of MS have been updated [153] and novel imaging patterns, such as the central vein sign, are explored as potential diagnostic biomarkers of the disease [154]. Additionally, the implementation of artificial intelligence applications, such as machine and deep learning for the differential diagnosis of demyelinating disorders, is currently under research [155]. Consequently, in the near future, automated analyses of advanced neuroimaging techniques are expected to significantly improve the diagnostic accuracy of various demyelinating disorders and aid clinicians in their differential diagnosis [156]. Images from MRIs performed in representative cases of aged people with secondary CNS demyelination, are depicted in Figure 1.

Despite all the recent technological advances, clinical history and examination are of cardinal importance in the diagnostic procedure of CNS demyelination. History of radiation, infection, vaccination, or treatment with certain agents, often accompanied by atypical findings that may constitute “red flags” for certain primary demyelinating disorders, may raise clinicians’ suspicions for secondary demyelinating disorders [157]. In the case of elderly patients, advanced age is often an independent “red flag” for primary demyelinating disorders such as MS [158].

## 7. Conclusions

Prompt recognition and diagnosis of conditions which cause secondary demyelination is crucial since many of these conditions are treatable and reversible. However, the occurrence of these conditions in the elderly poses additional challenges in their management, due to the significant alterations in the capacity of CNS repair mechanisms and other responses to damage [159]. Clinicians should be alert for all aforementioned demyelinating disorders in elderly individuals, since in a significant number of them, such as infections, immediate treatment approaches are required, while in other, such as in demyelination due to pharmaceutical agent administration, changes in a long-term disease management plan may be required.

## Figures and Tables

**Table 1:** Causes of secondary CNS demyelination in elderly people.

Causes of Secondary Demyelination in Elderly People	Common Clinical Manifestations	Treatment Options
**1.** **Demyelination due to infectious agents**		
*Progressive multifocal encephalopathy*	Encephalopathy, focal neurological deficits, cognitive deficits, seizures [27]	Various agents under Investigation [39]
*Tabes dorsalis*	Sensory ataxia, visual loss, spastic paraparesis, sensory loss [45]	Penicillin G, doxycycline, tetracycline, ceftriaxone [45,49,50]
**2.** **Postinfectious/postvaccinal demyelination**		
*Acute disseminated encephalomyelitis*/ *Postinfectious encephalitis*	Encephalopathy, focal neurological deficits, neuropsychiatric symptoms [57]	Steroids, plasmapheresis, intravenous immunoglobulin, cyclophosphamide [61]
*Postvaccinal transverse myelitis*	Spastic paraparesis, lower limp sensory loss [52]	Steroids, plasmapheresis, intravenous immunoglobulin [53,54]
**3.** **Vitamin deficiencies**		
*Subacute combined degeneration*	Neuropsychiatric symptoms, impaired proprioception and vibration [90]	Vitamin supplementation [91]
*Wernicke encephalopathy*	ophthalmoplegia, ataxia, confusion [97]	Thiamine [102]
*Marchiafava–Bignami syndrome*	spasticity, ataxia, seizures, unilateral apraxia, anomia, agraphia [110]	Vitamin supplementation, steroids [111]
**4.** **Osmotic demyelination syndromes**		
*Central pontine/extrapontine myelinolysis*	Encephalopathy, seizures, dysarthria, spastic quadriparesis, pseudobulbar paralysis, ataxia, lethargy [76]	Steroids, thyrotrophin releasing hormone, and intravenous immunoglobulin under investigation [77]
**5.** **Chemical agents and medical therapy**		
*Postradiation/drug related Demyelination*	Focal neurological deficits, cognitive disturbances [116]	Antioxidants and vitamins under investigation, drug interruption [114]
**6.** **Paraneoplastic Syndromes**		
*Paraneoplastic encephalitis*	Encephalopathy, seizures, neuropsychiatric symptoms, ataxia [142]	Tumor excision. Cyclophosphamide, rituximab, steroids, mycophenolate mofetil, and tacrolimus under investigation [143].

## Data Availability

Not applicable.
